# HLA-B∗44 Is Associated with Dengue Severity Caused by DENV-3 in a Brazilian Population

**DOI:** 10.1155/2013/648475

**Published:** 2013-06-02

**Authors:** Liciana Xavier Eurico de Alencar, Ulisses de Mendonça Braga-Neto, Eduardo José Moura do Nascimento, Marli Tenório Cordeiro, Ana Maria Silva, Carlos Alexandre Antunes de Brito, Maria da Paz Carvalho da Silva, Laura Helena Vega Gonzales Gil, Silvia Maria Lucena Montenegro, Ernesto Torres de Azevedo Marques

**Affiliations:** ^1^Virology and Experimental Therapy Laboratory, Aggeu Magalhães Research Center, Fiocruz, Avenida Moraes Rego s/n, Campus da UFPE, Cidade Universitária, 50670-420 Recife, PE, Brazil; ^2^Department of Electrical Engineering, Texas A&M University, 3128 TAMU, Station College Station, TX 77843-3128, USA; ^3^Department of Infectious Diseases and Microbiology, Center for Vaccine Research, University of Pittsburgh, 3501 Fifth Avenue, Biomedical Science Tower 3, Room 9022, Pittsburgh, PA 15261, USA; ^4^Central Laboratory of Public Health, Secretaria de Saúde do Estado de Pernambuco, Praça Oswaldo Cruz s/n, Boa Vista, 50050-210 Recife, PE, Brazil; ^5^Hospital das Clínicas, Universidade Federal de Pernambuco, Clínica Médica, Avenida Morais Rego s/n, Campus da UFPE, Cidade Universitária, 50670-420 Recife, PE, Brazil; ^6^Departamento de Bioquímica e Biofísica, Centro de Ciências Biológicas, Universidade Federal de Pernambuco, Avenida Moraes Rêgo 1235, Cidade Universitária, 50670-420 Recife, PE, Brazil; ^7^Departamento de Imunologia do Centro de Pesquisas Aggeu Magalhães-CPqAM, Fiocruz, Avenida Moraes Rego s/n, Campus da UFPE, Cidade Universitária, 50670-420 Recife, PE, Brazil

## Abstract

Human leukocyte antigen (HLA) alleles have been correlated with susceptibility or resistance to severe dengue; however, few immunogenetic studies have been performed in Latin American (LA) populations. We have conducted immunogenetic studies of HLA class I and II alleles in a cohort of 187 patients with DENV-3 infection and confirmed clinical diagnosis of either severe dengue, known as dengue hemorrhagic fever (DHF), or the less severe form, dengue fever (DF), in Recife, Pernambuco, Brazil. An association analysis was performed using Fisher's association test, with odds ratios (ORs) calculated using conditional maximum likelihood estimates. HLA-B∗44 (*P* = 0.047, OR = 2.025, 95% CI = 0.97–4.24) was found to be associated with increased susceptibility to DHF in response to DENV-3 infection. In addition, HLA-B∗07 (*P* = 0.048, OR = 0.501, one-sided 95% CI = 0–0.99) and HLA-DR∗13 (*P* = 0.028, OR = 0.511, one-sided 95% CI = 0–0.91) were found to be associated with resistance to secondary dengue infection by DENV-3. These results suggest that HLA-B∗44 supertype alleles and their respective T-cell responses might be involved in susceptibility to severe dengue infections, whereas the HLA-B∗07 supertype alleles and DR∗13 might be involved in cross-dengue serotype immunity.

## 1. Introduction

Dengue virus (DENV) has four serotypes, named as DENV-1, DENV-2, DENV-3 and DENV-4. The majority of dengue infections are subclinical; however, the clinical manifestations of dengue infection range from the benign, self-limited dengue fever (DF) to a vasculopathy syndrome known as dengue hemorrhagic fever (DHF) that can lead to hypovolemic dengue shock syndrome (DSS). It is often observed in epidemiological studies that the more severe illness occurs more frequently in secondary heterotypic dengue infection [1–7]. There are two main hypotheses to explain the higher frequency of DHF in secondary infections. The first is that heterotypic antibodies would bind to the dengue virus and would facilitate the viral entry into cells expressing Fc receptors; this theory is known as antibody dependent enhancement (ADE) [8, 9]. The second hypothesis is that anti-dengue memory T cells that cross-react with related but altered peptide epitopes would induce the T cells to produce abnormal levels of cytokines leading to vasculopathy. This theory is known as “original antigenic sin” (OAS) or “altered peptide ligand” (APL). The theories are not mutually exclusive, and both are based on the fact that previous dengue infection is a risk factor for developing more severe disease. However, 99% or more of the secondary dengue infections are benign, suggesting that host and virological genetic factors may be also involved.

The geographic distribution of the DENV and the incidence of the viral infection have grown fast in recent decades, and dengue fever is considered to be one of the most important reemergent tropical diseases [10]. However, there are significant differences in the clinical and epidemiological profile of dengue between the countries of Latin America (LA) and Southeast Asia (SA). In SA countries DHF cases are most common and the dengue morbidity and mortality are more prevalent in children under 15 years of age, whereas in LA countries most of the dengue cases are mild and manifest mostly in adults [11–14]. The differences in the age distribution between LA and SA can be attributed to the differences on the history of these epidemics in these regions and their age profiles are likely to converge over time [15]. However, the differences on the frequency of severe cases may be also partially explained by genetic differences between these populations. 

It has been proposed that a high prevalence of dengue resistance genes in the LA population could explain the differences on the frequency of severe cases between SA and LA [16, 17]. Host genetic polymorphisms involved in innate immune responses have been shown to be correlated with resistance to DHF, such as a variant of the FcGRIIA allele [18], functional polymorphisms of MBL2 [19], and the polymorphism of the CD209 promoter [20]. Similarly, studies of MHC-encoded transporters associated with antigen processing (TAP) genes have also shown associations with DHF [21, 22]. In addition, the analyses of tumor necrosis factor (TNF) and lymphotoxin alpha (LTA) genes have recently revealed specific combinations of TNF, LTA, and HLA class I alleles that associate with DHF and production of LTA and TNF [23]. 

Indeed, several aspects of T cell functionality are altered in DHF patients, including proliferation, activation status, production of cytokines, and their survival [4, 24–26]. All these functions are influenced by specific recognition, through T cell receptors (TCRs), of the antigen associated with HLA molecules. Thus, polymorphisms of HLA genes may also play an important role in dengue severity. Several genetic variations in HLA class I alleles have been found to correlate with dengue severity in Southeast Asian populations. For example, a protective role for HLA-A*33 in the ethnic Vietnamese [27] and HLA-A*0203 in the ethnic Thai population has been reported [28], as has a pathogenic role for HLA-A*24 in the Vietnamese [27] and HLA-A*0207 [28] and HLA-B*13 [29] in the Thai population. However, there have been fewer studies examining possible correlations between dengue severity and HLA variation in LA populations. In Mexico, LaFleur and colleagues (2002) reported that HLA-DRB1*08 and DRB1*04 were associated with susceptibility and resistance, respectively, to developing DHF [30]. HLA-A*1 and Cw1 were associated with shock, dehydration, and severe hemorrhage (DHF/dengue shock syndrome) in the Cuban population [31]. 

 Eighty percent of dengue cases reported in LA occur in Brazil, but studies correlating dengue severity and HLA alleles in this country are scarce. In order to determine if the Brazilian HLA allelic diversity plays a role in protection or susceptibility to DHF, we have analyzed the HLA-A, B, C, DR, and DQ genotypes of a cohort of dengue patients from Recife, Brazil. We found that patients bearing the HLA-B*44 allele had increased susceptibility to DHF. We also found that the alleles HLA-B*50 and -DR*16 were associated with increased risk for DHF, whereas HLA-DR*9 and -DR*12 were associated with protection against DHF, but these alleles were not sufficiently prevalent in the population (allelic frequency < 5%) for us to reach a significant conclusion. However, the allele HLA-B*50 is part of the HLA-B*44 supertype, lending support to the hypothesis that HLA-B*50 is in fact a risk factor for DHF. In addition, we found that individuals with the HLA-B*07 and HLA-DR*13 alleles appeared to be resistant to secondary infection by DENV-3.

## 2. Methods

### 2.1. Ethics Statement

This study was reviewed and approved by the Ethics Committee of the Brazilian Ministry of Health CONEP: 4909; Process number 25000.119007/2002-03; CEP 68/02. FWA00001515; IRB00001858. In addition, the Johns Hopkins University Institutional Review Board also reviewed and approved this study as protocol JHM-IRB-3: 03-08-27-01. Informed consent was obtained from all volunteers and all clinical investigation was conducted according to the principles expressed in the Declaration of Helsinki.

### 2.2. Study Design

A cohort of dengue patients admitted to hospitals in the city of Recife, state of Pernambuco, Brazil, was characterized. During this study there was a large outbreak of DENV-3 and most of our volunteers were of this serotype, so for this study we selected only individuals infected with DENV-3. This cohort has been characterized previously in [32, 33]. Briefly, multiple blood samples collected during the first 30 days after the appearance of the symptoms were used to obtain sera, plasma, and peripheral blood mononuclear cells (PBMC). All specimens were cryopreserved for further characterization of the cohort. Dengue diagnosis was performed using a combination of virus isolation in C6/36 cells [34] and RT-PCR [35] of samples collected on the day of admission (first sample), together with ELISAs to detect antidengue IgM and IgG in the first and follow-up samples.

 Dengue cases were confirmed by ELISA, RT-PCR, or virus isolation and were clinically classified according to the classic World Health Organization (WHO) criteria into two classes: dengue fever (DF) and dengue hemorrhagic fever (DHF) [36]. DF cases were characterized by high fever lasting for 7 days and accompanied by at least two of the following symptoms: severe headache, retroorbital pain, myalgia, arthralgia, and rash associated with platelet counts above 100,000/mm^3^. Dengue hemorrhagic fever cases were defined as having the same clinical manifestations as for DF, but with evidence of hemorrhage, thrombocytopenia (platelets < 100,000/mm^3^), and plasma leakage following defervescence. The classification of primary and secondary dengue infections was based on the kinetics of the IgM and IgG response, according to validated criteria published previously [32]: primary infection (P) was defined as the absence of specific antidengue IgG antibodies in the first serum samples during the acute phase, with IgM, virus, and/or viral RNA being detected and followed by the presence of antidengue IgG in convalescence serum samples. Secondary infection (S) was defined as the detection of specific antidengue IgG in the first acute sample and the absence of antidengue IgM, associated with a positive RT-PCR and/or virus isolation. With the exception of a few cases, antidengue IgM was present in the convalescent serum samples.

### 2.3. DNA Extraction

Genomic DNA was extracted from frozen PBMC samples using the PureLink Genomic DNA (Invitrogen) kit, according to the manufacturer's instructions. The cells were digested with Proteinase K at 55°C using an optimized digestion buffer formulation that aids in protein denaturation and enhances Proteinase K activity. Any residual RNA was removed by digestion with RNAse A prior to binding samples to the silica membrane. The lysate was mixed with ethanol and PureLink Genomic Binding Buffer that allowed high DNA binding PureLink Spin Column. The DNA bound to the silica-based membrane in the column and impurities were removed by thorough washing with Wash Buffers. The genomic DNA was then eluted in low salt Elution Buffer. The isolated DNA was 20–50 kb in size and was suitable for PCR. The samples were stored at −20°C and later used for HLA typing.

### 2.4. HLA Typing

Extracted DNA was used for low-resolution HLA typing carried out by PCR with Sequence Specific Primers amplification methods (Commercial kit Invitrogen SSP UniTray) as previously described [37, 38]. Setup included mixing a reaction buffer with human genomic DNA sample and Taq DNA Polymerase, dispensing the mixture to the UniTray sealing and then performing thermal cycling. After cycling was completed the PCR products were loaded onto a 2% (w/v) agarose gel for electrophoresis. Next the gel was stained with ethidium bromide, photographed using a gel documentation system UV transilluminator, and analyzed with UniMatch Plus software in order to determine the HLA-A, HLA-B, HLA-Cw, HLA-DR, and HLA-DQ loci using a worksheet for the specific amplification patterns.

### 2.5. Statistical Analysis

In order to analyze the potential associations between different groups (primary versus secondary infections; DF versus DHF), we determined the frequency of a given allele in each of the groups under analysis. We then applied the two-sided Fisher's test on each allele, in order to verify the possibility of significant differences between the frequencies of DF versus DHF. For the comparison between frequencies of primary and secondary infections we employed a one-sided Fisher's test for OR < 1, as the only effect of interest (protection against secondary infection). The reported OR and associated confidence interval limits in both cases are estimated using the conditional maximum-likelihood (CML) method. No correction for multiple testing was attempted, implying that further validation of the results with additional patients in follow-up studies is recommended. In this regard, significant alleles with an allelic frequency inferior to 5% need to be appreciated with caution.

## 3. Results

### 3.1. Validation of the Genotyping Data

The HLA type of the HLA-A, HLA-B, HLA-C, HLA-DR, and HLA-DQ loci of the 187 confirmed dengue of DENV-3 genotype III [32] was determined in low resolution. The summary of the demographic profile of the patient volunteers in this study is shown on Table 1 and the complete table is shown in supplemental material Table S1 (see Supplementary Materials available online at http://dx.doi.org/10.1155/2013/648475). 

**Figure 1:**
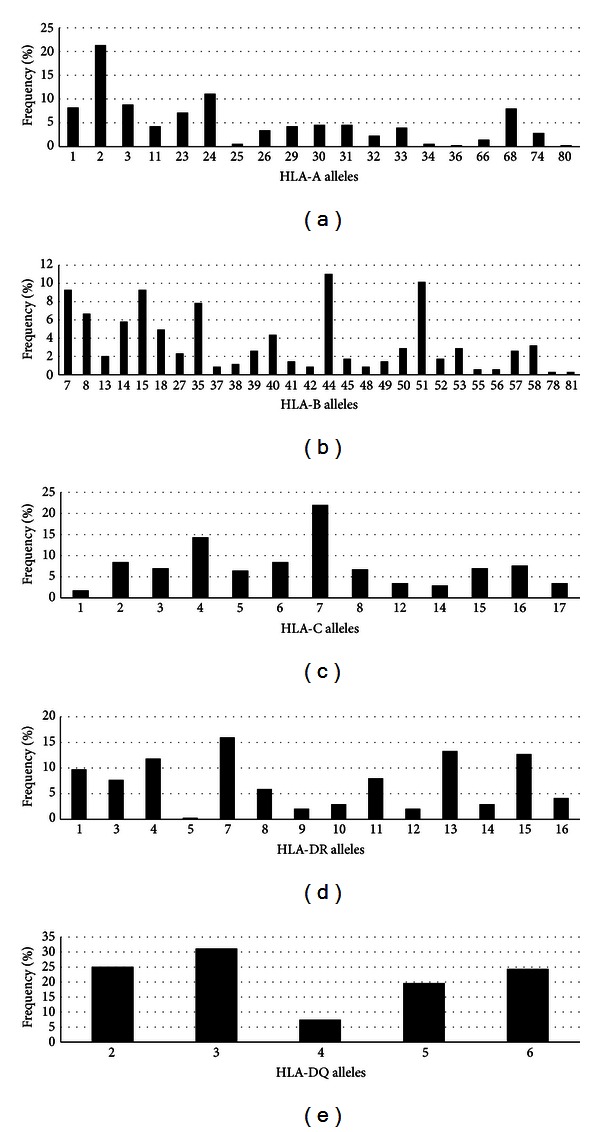
General frequency for HLA in the study population. (a) HLA-A, (b) HLA-B, (c) HLA-C, (d) HLA-DR, and (e) HLA-DQ.

The quality and representativeness of the HLA genotyping data of our dengue cohort were initially accessed by comparing the frequency of the alleles identified to those found in two samples of Brazilian populations of normal blood bank volunteers deposited in the PubMed's MHC database (dbMHC) (http://www.ncbi.nlm.nih.gov/mhc/): one population was from the state of Minas Gerais (designated here as data set dbMHC1) and another from the state of São Paulo (designated dbMHC2). The dbMHC1 data set contains only HLA-A and HLA-B genotypes, whereas the dbMHC2 dataset contains HLA-A, HLA-B, HLA-C, and HLA-DR genotypes. These databases are considered to represent the HLA diversity of these populations [39, 40]. High-resolution genotyping information in the dbMHC data sets was ignored, and alleles that were not carried by any of the patients were also ignored, so as not to artificially inflate the correlation. In Table 2 it is presented the results with the correlation coefficients, confidence intervals, and significance. These analyses indicated that there was a high correlation between the observed allelic frequencies in our cohort named LaViTE and both dbMHC1 and dbMHC2 data sets indicating that our sample does not have significant differences on the HLA profile from these other two Brazilian groups. This result indicates that the genotype of the population making up our cohort is representative of the Brazilian genetic background for all the available MHC genes investigated here. The relative frequencies of the alleles studied in the various data sets are shown in Figure 1.

### 3.2. Association between HLA Allele and Dengue Infection History

**Figure 2:**
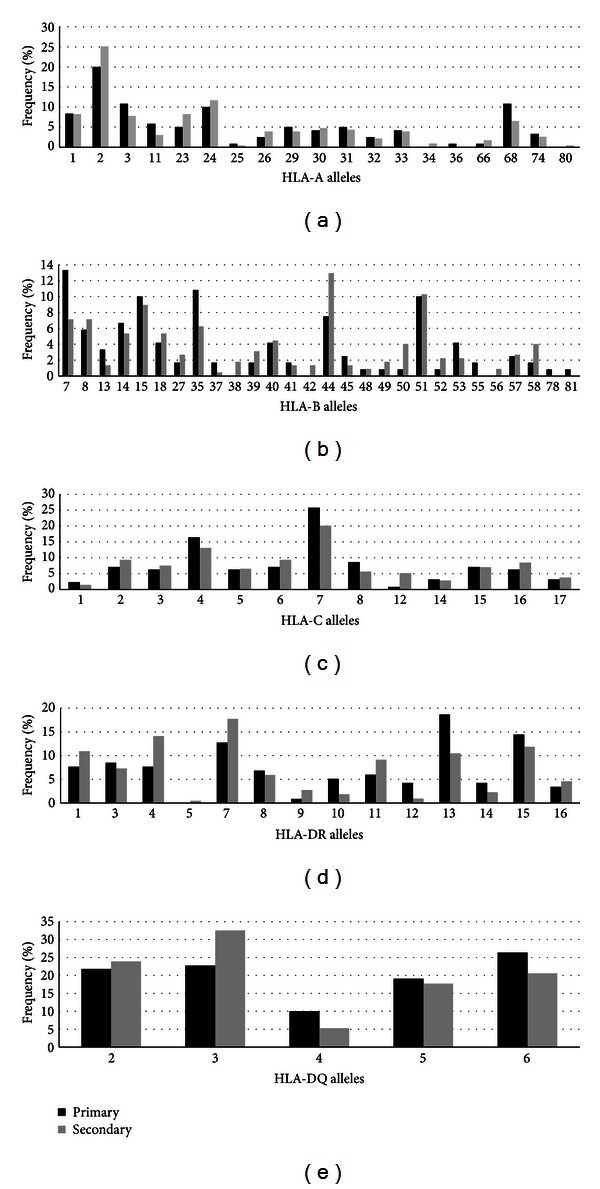
Allele versus primary-secondary frequency for HLA in the study population. (a) HLA-A, (b) HLA-B, (c) HLA-C, (d) HLA-DR, and (e) HLA-DQ.

It is postulated that CD4 and CD8 T cell responses play a role in protection against flavivirus and this has been shown in epidemiological studies [41] and in animal models [42], although the requirements for T cell mediated protection is not known. HLA alleles can bind to different peptide repertoires and some are more permissive to a wider range of peptides than others [43]. In addition, some of the peptides epitopes can be more conserved across serotypes than others. It is also known that dengue infection by one serotype leads to cross-serotype immunity that can last for several months and this protection is thought to be mediated by T cells [44, 45]. Based on this, we postulated that some HLA alleles would be more capable of producing cross-serotype T cell responses and if so the presence of this allele could associate to reduced susceptibility to secondary infections. To test this hypothesis we investigated the associations between dengue infection history (primary X secondary) and the HLA allelic frequency. Sixty-six (35.3%) of the cases were diagnosed as having primary infections (“Prim,” Table 1) and 121 (64.7%) as having secondary infections (“Sec,” Table 1). A one-sided Fisher's association test was used to identify differences in the distribution of each allele of a given HLA haplotype with regard to the type of infection, whether primary or secondary (Table 3). The one-sided test seeks to identify OR < 1, as the only effect of interest in this case (protection against secondary infection). According to this association test, two alleles, HLA-B*07 (*P* = 0.048, OR = 0.501, one-sided 95% CI = 0–0.99) and HLA-DR*13 (*P* = 0.028, OR = 0.511, one-sided 95% CI = 0–0.91), were found to be significantly associated at the 95% level. Among patients with the HLA-B*07 genotype, 13.33% were characterized as having primary infection, whereas 7.14% had a secondary infection, suggesting that this allele might be involved in eliciting cross-serotype protective CD8^+^ T cell responses. Similarly, among dengue patients with the HLA-DR*13 genotype, 18.64% were characterized as having primary infection, whereas 10.45% had a secondary infection, suggesting that this allele is also likely to elicit cross-protective responses against heterotypic dengue serotypes.  Figure 2 shows comparison of the frequency of all the alleles studied, with regard to the type of infection. Although these results are statistically significant these associations need to be further validated in larger cohorts.

### 3.3. Association between HLA Haplotype and Dengue Severity

**Figure 3:**
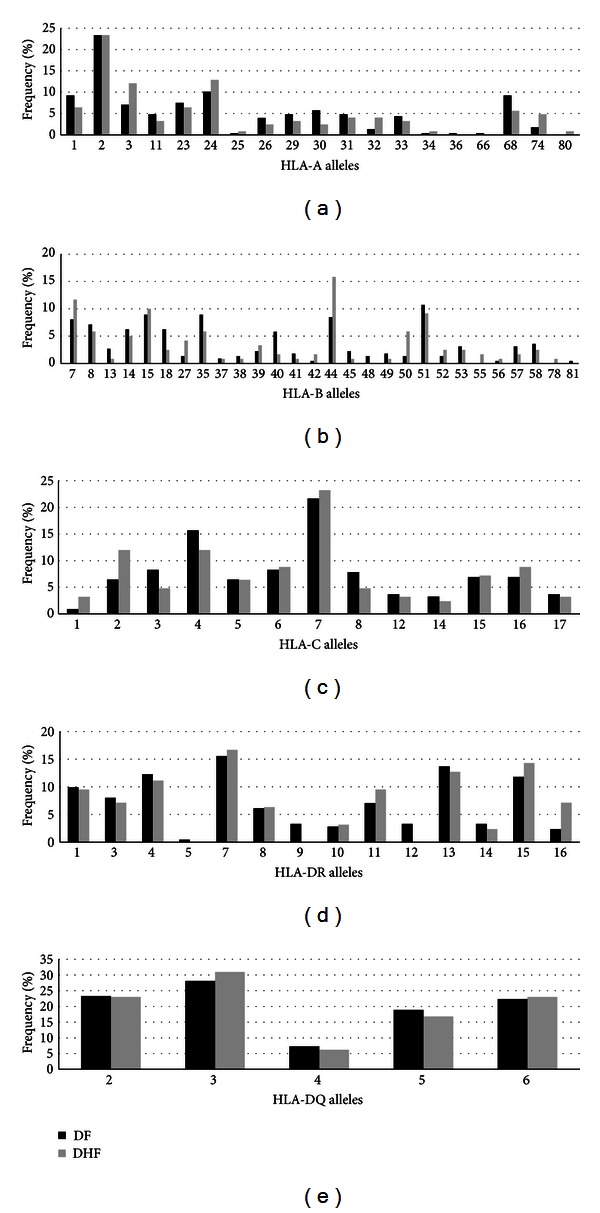
Allele versus DF-DHF frequency for HLA in the study population. (a) HLA-A, (b) HLA-B, (c) HLA-C, (d) HLA-DR, and (e) HLA-DQ. DF: dengue fever; DHF: dengue hemorrhagic fever.

 T cell responses have been associated with both protection and also severity of the disease [46–48] and several HLA alleles have been associated with protection and susceptibility to severe disease [27–29]. So, we next examined the potential association between HLA allele and clinical outcome in 120 (64%) DF patients and 67 (36%) DHF patients. Taking the same approach that we had used for our analysis of the type of infection, we used Fisher's test to determine the difference in the distribution of each allele of a given HLA allele between DF and DHF patients (Table 4). The alleles that showed significant association with increased risk for DHF at the 95% level were B*44 (*P* = 0.047, OR = 2.025, 95% CI = 0.97–4.24), B*50 (*P* = 0.037, OR = 4.542, 95% CI = 1.01–27.72), and DR*16 (*P* = 0.046, OR = 3.173, 95% CI = 0.93–12.34). In addition, the alleles DR*09 and DR*12 (both with *P* = 0.049, OR = 0, 95% CI = 0–1.15) appeared to have a protective effect against DHF. However, the alleles B*50, DR*16, and especially DR*9 and DR*12 were all rare in the studied population, with an overall allelic frequency of less than 5%, indicating that the results for these alleles need to be appreciated with caution. The B*44 allele is a very common HLA-B allele in our study population (11% frequency) and, according to the results, its frequency is nearly twice as high in DHF patients (15.83%) as in DF patients (8.48%, Figure 3), reason why confidence is increased that this result is a true positive association. The allele B*50 was reported in 5.83% of the DHF cases and only in 1.34% of the DF cases. HLA-B*50 encodes a protein that has a similar peptide binding profile as the HLA-B*44, being considered a member of the B44 supertype; this lends support to the hypothesis that the repertoire of T cell responses elicited by the B*44 and B*50 alleles may be involved in the DHF pathogenic mechanism. Allele DR*16 was reported in 7.14% of the DHF cases and only in 2.36% of the DF cases, also suggesting an increased susceptibility to DHF. By contrast, the HLA-DR*9 and DR*12 genotypes were found to be protective; both had a 3.3% frequency in DF patients, whereas no DHF patients had the DR*9 and DR*12 genotypes.

## 4. Discussion

In this study we have genotyped HLA-A, HLA-B, HLA-Cw, HLA-DR, and HLA-DQ loci of 187 well-characterized dengue patients and this is the largest immunogenetic study performed in LA population reported to date. The analysis identified associations of HLA-B*44, -B*50, and -DR*16 alleles with susceptibility to DHF, whereas HLA-DRB1*09 and -DR*12 were associated with protection to DHF. In addition, the HLA-B*07 and -DR*13 were associated with lower frequency of secondary dengue infections suggesting that these HLA alleles might be involved in presenting protective cross-reactive T cell epitopes.

Dengue T cell responses have been correlated with dengue protection and pathogenesis and are considered as a double-edged sword. Immunological studies have shown that DHF patients present decrease in T cell proliferation and IFN*γ* production before defervescence [24]. In addition, increased levels of activated T cells [49], expressing cytokines [25] and undergoing apoptosis, are also observed during the acute phase of dengue infection [4, 26]. All of these functions are thought to be dependent upon specific recognition, through T cell receptors (TCRs), of particular antigens associated with HLA molecules. Indeed, CD8^+^ T cells-specific response against a dengue NS3 epitope has been associated with disease severity; however, the mechanisms of specific T cell responses on pathogenesis are not understood [46]. Several studies have shown statistically significant associations between dengue disease severity and specific HLA molecules [50]. Thus, HLA peptide epitope repertoire may be a factor determining patient outcomes in the case of dengue infection either by eliciting protective T cell responses against cross-serotype conserved sequences [51] or by eliciting T cell responses to variable epitopes that can lead to APL or OAS responses [4, 52].

Four immunogenetic studies have explored HLA association with dengue severity in LA populations [30, 31, 53, 54]. Paradoa Pérez et al. have found that A*01 was associated to susceptibility DHF, whereas A*29 was associated with resistance in a cohort of 82 patients [31] in Cuba. Sierra and colleagues (2007) studied a cohort of 120 dengue patients and found that A*31 and B*15 were associated with susceptibility to DHF and DRB1*04 and DRB1*07 were associated with resistance to DHF [54]. Consistent with Sierra study, LaFleur et al. also found that DRB1*04 was associated with resistance to DHF in a Mexican cohort of 47 patients [30]. In our study we did not find an association between A*01 (Figure 3) and DHF, and we found a slightly lower frequency of A*29 and A*31 in DHF patients; these alleles had a very low frequency in our cohort and the frequencies were not statistically significant in DF from DHF. However, we observed a larger frequency of B*15 in DHF, but it was not significant. We did not observe any protective effect of DRB1*04 or *07 in our cohort. However, here are many methodological differences among all these studies to allow a comprehensive comparison and these differences together with other environmental and epidemiological differences can explain the different findings in the different studies. To date, there have been no reports regarding potential correlations between dengue severity and HLA alleles in Brazil. In the only study performed in this context in a Brazilian population, the authors compared polymorphisms in HLA classes I and II in DF patients and healthy people, associating HLA-DQ1 with susceptibility to dengue infection [53]. 

Similar immunogenetic studies performed have been performed in SA. However, the size of the study cohorts was in general significantly larger than the ones in LA. Chiewsilp et al. [29] found that A*02 was associated with susceptibility to DHF, whereas B*13 was found associated with resistance. In a larger study in a Thai population of 263 dengue patients, Stephens and colleagues (2002) [28] also found an association with A*02; however, in this study they used high resolution HLA genotyping and identified the association to be with the A*0207 subgenotype and was specific for DENV-2. In addition, they also found that HLA A*11, B*51, and B*46 were associated with susceptibility to DHF, whereas the HLA B*15 and B*44 were associated with resistance to secondary DHF infections. In fact, the same HLA B*44 was associated with increased risk in patients with primary DHF infections in Thai population. The B*44 association with resistance to DHF in secondary infections and susceptibility to DHF in primary infection is intriguing. It suggests that studies about the role of HLA alleles in dengue disease need to take in consideration the serotype and the order of infection. In subsequent study and in a larger Thai group of 435 dengue patients Vejbaesya and colleagues (2009) [23] found that HLA B*48 was associated with DHF, however the previous associations found were not confirmed. In a Vietnamese study with 352 dengue patients Loke and colleagues (2001) [27] identified correlations between HLA A*24 and susceptibility to DHF grades III and IV and correlation between A*33 and resistance to DHF grades III and IV. In other Vietnamese study [55] was reported HLA association between the HLA A*24 allele variant with a histidine at position 70 and dengue shock in primary infections, whereas the HLA DRB1*0901 was found associated with resistance to dengue shock syndrome as result of DV2. The results presented here are consistent with the associations found by Nguyen and colleagues (2008) [55]; we also found that the DRB1*09 is associated with protection to DHF. The results of our study were also consistent with the ones of Chiewsilp and colleagues (1981) [29]; there was a slightly higher frequency of A*02 in DHF patients as compared to DF and we also observed that the B*13 allele was more frequent in DF than in DHF; however, the frequency of the B*13 allele in our cohort is low and neither of these differences was statistically significant. As the study of Stephens and colleagues (2002) [28], we found highly significant associations between B*44 with the severity of the dengue disease. In our study, the allele B*44 was associated with susceptibility to DHF and in the associations reported by Stephens the B*44 showed susceptibility to DHF only in primary infections. In a more recent and very elegant study performed in a Sri Lankan cohort of 110 DHF patients, and a control group of 119 individuals who had never reported a dengue fever was found that HLA-A*31 and DRB1*08 were associated with susceptibility to DHF [56]. In our study we did not find differences in the HLA frequencies of our dengue cohort and the general population. However in our cohort, we only have DF and DHF grades I and II, and our control population is random and does not allow a similar type of comparison. 

In general, several HLA alleles have been associated with resistance and susceptibility to DHF; however, the results are not consistent and still lack a proper demonstration of cause and effect. Of course, it is clear that multiple factors are involved in the pathogenesis of DHF. The findings presented here show a significant association of the HLA-B*44, -B*50, and -DR*16 alleles with increased susceptibility to DHF and represent another attempt to improve our understanding of the role of HLA molecules on outcome of DENV infection. However, it is important to perform additional genetic studies in larger dengue cohorts.

## Supplementary Material

Demographic, clinical and HLA typing data for all 187 subjects analyzed in this study.

## Figures and Tables

**Table 1:** Distribution of DENV-3 cases with regard to demographic profiles, clinical manifestation, and type of infection.

Clinical diagnosis	Median age (range)	Sex		IgM (+)	IgG (+)	Type of infection		Total
		Male	Female			Primary	Secondary	
DF	27 (4–76)	61	59	79	75	47	73	120
DHF	17 (0,6–84)	25	42	53	48	19	48	67

DF: dengue fever; DHF: dengue hemorrhagic fever; IgM: immunoglobulin M; IgG: immunoglobulin G.

**Table 2:** Correlations between the observed allelic frequencies in the LaViTE cohort and two other Brazilian datasets deposited in the dbMHC database of the NCBI, dbMHC1 (Minas Gerais, Brazil), and dbMHC2 (São Paulo, Brazil).

HLA loci	Correlation	95% CI	*P* value
HLA-A			
LaViTE, dbMHC1	0.9776	0.9415–0.9915	<10^−12^
LaViTE, dbMHC2	0.9445	0.8588–0.9788	<10^−8^
HLA-B			
LaViTE, dbMHC1	0.9171	0.8357–0.9591	<10^−12^
LaViTE, dbMHC2	0.8223	0.6640–0.9100	<10^−8^
HLA-C			
LaViTE, dbMHC2	0.9114	0.7374–0.9719	<10^−5^
HLA-DR			
LaViTE, dbMHC2	0.7977	0.4401–0.9368	<0.05

HLA: human leukocyte antigen; CI: confidence interval.

**Table 3:** Results of one-sided Fisher's test (OR < 1) of association between the various HLA alleles analyzed and the type of dengue infection (primary versus secondary infections).

HLA allele	Frequency (%)		Fisher's test	
	Primary	Secondary	OR (95% CI)	*P*
B07	13.33	7.14	0.501 (0–0.99)	0.048
DR13	18.64	10.45	0.511 (0–0.91)	0.028

HLA: human leukocyte antigen; OR: odds ratio; CI: confidence interval; *P*: *P* value.

**Table 4:** Results of two-sided Fisher's test of association between the HLA alleles analyzed and the clinical manifestations of dengue (DHF versus DF).

HLA allele	Frequency (%)		Fisher's test	
	DF	DHF	OR (95% CI)	*P*
B44	8.48	15.83	2.02 (0.97–4.24)	0.047
B50*	1.34	5.83	4.54 (1.01–27.72)	0.037
DR9*	3.3	0	0 (0–1.15)	0.049
DR12*	3.3	0	0 (0–1.15)	0.049
DR16*	2.36	7.14	3.17 (0.93–12.34)	0.046

HLA: human leukocyte antigen; DF: dengue fever; DHF: dengue hemorrhagic fever; OR: odds ratio; CI: confidence interval; *P*: *P* value.

Alleles with overall allelic frequency inferior to 5% are indicated with a star.
