# Supplementary material for: HLA-B∗44 Is Associated with Dengue Severity Caused by DENV-3 in a Brazilian Population
Source: J Trop Med. 2013 Jun 2;2013:648475. doi: 10.1155/2013/648475 (PMC3684019; doi:10.1155/2013/648475)
Supplement: Supplementary file 1 — Demographic, clinical and HLA typing data for all 187 subjects analyzed in this study. [file 648475.f1.pdf]

Supplemental Material

S1. Demographic, clinical data and HLA typing for all patients infected with dengue virus.

Patient ID	Age	Sex	Type of Infection	Clinical Diagnosis	Human Leukocyte Antigen (HLA)									
					A*	AA*	B*	BB*	C*	CC*	DRB1*	DDRB1*	DQB1*	DDQB1*
P083	36	M	Sec	DF	2	24	8	15	ND	ND	13	15	6	0
P086	36	F	Prim	DF	1	26	40	58	3	7	11	13	3	6
P087	23	M	Sec	DF	23	24	40	51	2	14	8	11	3	4
P094	28	F	Prim	DHF	24	ND	14	ND	2	8	1	10	5	ND
P099	35	M	Sec	DF	2	23	44	48	4	0	4	7	2	3
P102	21	F	Prim	DHF	1	74	7	57	6	7	7	15	2	6
P104	66	F	Sec	DHF	24	34	14	27	1	8	3	4	2	3
P108	40	M	Sec	DF	1	24	35	44	4	0	4	15	3	6
P111	21	F	Prim	DHF	2	23	35	44	2	4	8	13	4	6
P113	27	M	Sec	DF	24	29	14	44	2	16	1	7	2	5
P114	31	M	Prim	DF	1	3	14	51	8	15	4	0	3	0
P117	44	F	Sec	DF	23	31	8	40	3	0	1	4	3	5
P119	68	M	Sec	DF	2	31	40	44	3	16	4	0	3	0
P125	84	F	Sec	DHF	2	23	7	49	7	0	8	13	3	6
P126	14	M	Sec	DF	2	30	ND	ND	6	17	4	11	3	5
P127	48	F	Prim	DF	2	32	15	40	3	5	4	10	3	5
P128	26	F	Prim	DHF	3	32	37	40	2	6	10	13	5	6
P129	29	F	Sec	DF	1	3	35	58	4	6	11	0	3	6
P130	18	M	Sec	DF	ND	ND	37	40	2	6	10	13	5	6
P132	27	F	Prim	DF	2	0	14	15	3	8	7	8	2	4
P135	34	M	Sec	DF	2	3	38	51	12	16	7	16	3	5
P136	27	F	Prim	DF	2	68	15	53	2	4	1	7	2	0
P145	19	M	Prim	DHF	3	66	35	58	4	7	13	15	6	0
P155	26	F	Sec	DF	2	23	45	58	6	0	1	15	5	6
P187	35	F	Sec	DF	2	26	7	38	7	12	3	15	2	6

S1. Cont.

Patient ID	Age	Sex	Type of Infection	Clinical Diagnosis	Human Leukocyte Antigen (HLA)									
					A*	AA*	B*	BB*	C*	CC*	DRB1*	DDRB1*	DQB1*	DDQB1*
P189	37	M	Sec	DF	30	68	35	53	ND	ND	8	11	4	6
P193	68	F	Sec	DHF	3	11	44	58	5	6	3	4	3	4
P198	43	M	Sec	DF	3	68	7	18	7	8	11	15	3	6
P201	60	F	Sec	DF	11	68	51	52	4	12	4	0	3	0
P203	26	F	Sec	DHF	11	0	39	52	3	12	4	8	3	4
P204	29	M	Sec	DF	23	33	14	18	8	0	7	13	2	6
P205	30	M	Prim	DF	24	68	7	15	7	0	3	4	2	3
P206	36	M	Sec	DHF	2	24	27	35	1	4	1	11	3	5
P210	20	F	Prim	DF	23	29	44	49	7	16	13	15	6	0
P215	52	F	Prim	DF	24	33	8	44	2	7	3	13	2	6
P220	18	F	Sec	DF	2	30	8	18	7	0	3	11	2	3
P224	34	F	Prim	DF	2	29	7	44	7	16	1	15	5	6
P226	4	M	Prim	DF	2	0	7	51	7	15	1	11	3	5
P235	35	F	Sec	DF	2	3	35	51	4	16	13	0	6	0
P241	40	F	Sec	DF	2	26	7	49	7	0	9	10	2	5
P246	31	M	Prim	DF	24	0	8	14	7	8	3	0	2	0
P248	45	F	Sec	DF	2	0	35	40	3	4	ND	ND	3	5
P249	43	M	Prim	DF	11	30	18	35	4	5	3	4	2	3
P250	12	F	Prim	DF	2	30	18	35	4	5	3	13	2	5
P255	48	M	Sec	DF	2	31	15	40	2	3	1	4	3	5
P256	57	F	Sec	DF	2	32	40	44	3	16	13	15	6	0
P259	30	F	Prim	DF	29	68	15	35	2	4	1	12	5	0
P260	44	F	Prim	DHF	3	68	7	53	4	7	13	15	6	0
P263	25	F	Prim	DF	3	74	51	81	8	15	12	15	5	6
P265	36	F	Prim	DF	1	68	15	48	4	7	13	14	3	6

S1. Cont.

Patient ID	Age	Sex	Type of Infection	Clinical Diagnosis	Human Leukocyte Antigen (HLA)									
					A*	AA*	B*	BB*	C*	CC*	DRB1*	DDRB1*	DQB1*	DDQB1*
P266	48	M	Sec	DF	2	26	18	51	7	14	1	16	5	0
P267	33	M	Prim	DF	2	33	14	18	5	8	1	10	5	0
P270	23	M	Sec	DF	29	68	39	44	7	16	4	7	2	3
P275	57	F	Prim	DF	1	33	8	14	7	8	7	0	3	6
P276	45	M	Sec	DF	1	24	51	57	2	7	7	16	3	5
P277	41	M	Sec	DHF	30	74	42	0	7	17	8	15	3	6
P278	76	F	Sec	DF	2	26	8	51	7	14	7	11	2	6
P279	17	F	Prim	DF	2	68	7	35	4	7	13	16	6	0
P281	35	F	Sec	DF	24	33	14	18	8	12	1	11	3	5
P282	22	F	Prim	DF	23	68	51	53	6	16	7	13	2	6
P284	36	M	Sec	DF	3	24	15	35	3	4	3	8	2	4
P285	45	M	Sec	DF	3	26	35	51	2	4	7	11	2	3
P286	49	F	Sec	DF	33	68	14	53	4	8	9	13	6	0
P289	25	F	Prim	DF	1	3	7	35	3	15	7	15	2	5
P291	66	F	Sec	DF	24	31	40	45	3	15	4	13	3	0
P292	25	M	Prim	DF	26	68	14	53	4	8	4	13	4	6
P294	15	F	Prim	DF	24	31	40	52	3	15	4	8	3	4
P295	18	M	Prim	DF	2	11	18	35	4	7	11	15	3	5
P297	18	M	Prim	DF	33	68	35	44	4	5	13	15	6	0
P298	22	M	Sec	DF	2	68	44	57	5	12	7	15	3	6
P299	56	M	Prim	DF	1	24	37	51	6	15	10	15	5	6
P300	17	M	Prim	DF	11	36	35	53	4	0	7	14	2	5
P301	30	F	Sec	DF	2	23	14	41	2	17	1	4	3	5
P305	35	M	Prim	DHF	2	24	7	35	4	7	15	0	6	0
P306	47	F	Sec	DF	1	2	41	51	14	17	3	0	2	0

S1. Cont.

Patient ID	Age	Sex	Type of Infection	Clinical Diagnosis	Human Leukocyte Antigen (HLA)									
					A*	AA*	B*	BB*	C*	CC*	DRB1*	DDRB1*	DQB1*	DDQB1*
P307	41	F	Prim	DHF	2	24	7	15	7	14	11	14	3	0
P308	42	M	Sec	DF	23	33	13	41	6	17	7	0	2	0
P310	30	F	Sec	DF	3	74	15	35	4	5	13	15	6	0
P311	33	M	Sec	DF	2	24	44	57	4	6	3	14	2	5
P314	31	F	Sec	DF	1	2	52	57	7	15	4	13	3	6
P316	31	F	Sec	DF	2	30	18	35	4	5	1	3	2	5
P319	40	M	Prim	DF	2	11	13	15	6	14	7	14	2	3
P320	49	F	Sec	DF	2	30	13	0	6	0	7	0	2	0
P322	48	M	Sec	DF	2	3	7	58	6	7	7	15	2	6
P329	47	M	Sec	DF	3	24	8	49	7	0	3	13	2	3
P330	40	F	Sec	DF	2	74	39	57	2	12	1	0	5	0
P331	45	M	Sec	DF	11	68	27	53	2	4	9	11	3	0
P332	34	F	Sec	DF	2	11	7	44	5	7	8	12	3	4
P339	61	F	Prim	DHF	2	32	8	51	7	14	3	0	2	3
P348	46	F	Sec	DF	1	2	7	18	7	0	11	0	3	5
P355	58	M	Prim	DF	1	23	ND	ND	7	17	3	4	2	3
P358	26	M	Prim	DF	11	0	39	51	2	7	7	0	2	4
P361	15	M	Sec	DF	2	23	35	50	4	5	13	0	3	6
P363	5	F	Prim	DF	ND	ND	27	35	1	4	1	12	3	5
P370	35	M	Prim	DHF	23	0	44	55	1	4	11	15	3	6
P378	11	M	Sec	DF	23	29	14	44	8	17	1	7	2	5
P420	37	F	Sec	DHF	1	80	8	14	7	8	1	1	5	ND
P428	69	M	Sec	DHF	2	3	15	51	2	15	1	15	5	6
P430	25	F	Prim	DHF	2	24	8	44	5	7	13	16	ND	ND
P538	46	M	Sec	DF	23	26	ND	ND	4	7	9	12	3	0

S1. Cont.

Patient ID	Age	Sex	Type of Infection	Clinical Diagnosis	Human Leukocyte Antigen (HLA)									
					A*	AA*	B*	BB*	C*	CC*	DRB1*	DDRB1*	DQB1*	DDQB1*
P543	29	F	Sec	DHF	2	29	27	44	2	16	7	15	2	5
P549	10	F	Sec	DHF	2	68	7	35	4	7	3	8	2	3
P557	76	F	Sec	DHF	1	31	8	39	7	12	1	11	5	ND
P564	38	F	Prim	DHF	3	31	15	44	1	7	7	16	2	3
P603	25	F	Prim	DHF	2	ND	7	35	4	7	8	15	3	6
P613	39	F	Prim	DHF	2	24	7	51	7	15	13	15	6	ND
P633	39	F	Prim	DF	ND	31	15	57	7	8	4	15	3	3
P635	30	F	Prim	DHF	3	74	7	ND	7	17	3	11	3	4
P641	7	F	Prim	DF	23	31	7	15	7	0	7	8	2	4
P645	40	F	Sec	DHF	2	ND	44	51	5	15	8	11	3	4
P681	36	F	Prim	DF	2	ND	13	44	5	6	4	7	2	3
P718	14	M	Prim	DHF	1	24	8	55	3	7	7	14	2	5
P721	6	M	Prim	DF	2	33	41	45	16	17	3	ND	4	6
P749	5	M	Sec	DF	29	30	42	44	16	17	3	ND	4	6
P750	5	F	Prim	DF	2	74	15	45	4	ND	8	13	3	4
P751	25	F	Prim	DF	30	31	39	ND	7	15	8	15	4	6
P753	12	F	Sec	DF	1	23	7	8	3	7	7	15	2	6
P761	11	F	Sec	DHF	24	68	13	52	6	15	4	0	2	5
P764	9	F	Prim	DF	2	68	45	51	16	15	10	13	5	6
P765	30	F	Sec	DF	2	68	50	51	6	15	8	13	3	6
P767	10	M	Sec	DHF	24	26	14	44	2	5	7	10	2	5
P770	10	M	Prim	DF	3	30	ND	ND	4	5	13	0	6	ND
P775	12	M	Sec	DF	23	66	44	58	4	6	7	15	2	6
P776	7	M	Sec	DF	1	26	8	38	7	12	7	0	2	3
P778	7	M	Sec	DF	1	2	8	0	7	ND	3	0	2	ND

S1. Cont.

Patient ID	Age	Sex	Type of Infection	Clinical Diagnosis	Human Leukocyte Antigen (HLA)									
					A*	AA*	B*	BB*	C*	CC*	DRB1*	DDRB1*	DQB1*	DDQB1*
P784	7	F	Sec	DF	2	11	15	51	3	15	4	9	3	0
P788	11	F	Sec	DF	2	29	15	58	6	8	11	13	3	6
P790	6	M	Sec	DF	24	31	40	51	15	0	4	0	3	0
P799	12	M	Prim	DF	2	68	51	ND	4	16	13	0	3	5
P801	6	M	Sec	DF	2	30	15	35	4	14	14	0	3	0
P804	-	M	Sec	DF	24	31	39	ND	3	7	4	8	3	4
P805	8	M	Prim	DF	11	25	7	18	7	12	15	16	5	6
P807	10	F	Prim	DF	2	29	51	ND	4	14	8	13	4	6
P809	5	M	Prim	DF	1	11	13	57	6	ND	7	0	2	ND
P811	6	F	Sec	DF	2	24	27	58	7	0	4	7	2	3
P820	9	F	Sec	DF	2	3	48	51	4	15	1	4	3	5
P821	8	M	Sec	DF	1	2	8	14	7	8	1	7	2	5
P826	8	M	Sec	DF	24	34	18	44	5	ND	3	4	2	3
P827	12	F	Prim	DF	68	ND	14	40	3	8	1	0	5	ND
P828	12	M	Sec	DF	1	2	15	58	3	4	13	0	3	6
P829	12	F	Prim	DF	3	29	7	13	6	7	1	9	2	5
P830	6	M	Prim	DF	24	68	8	51	7	16	7	0	2	ND
P833	13	F	Sec	DF	24	ND	8	50	6	7	7	13	2	ND
P835	9	F	Prim	DF	30	31	ND	ND	3	8	12	12	2	5
P842	12	M	Sec	DF	2	33	15	56	1	2	1	7	2	5
P846	13	M	Sec	DF	1	24	8	15	7	ND	14	15	3	6
P847	8	M	Sec	DF	30	32	7	8	7	0	14	15	6	ND
P850	11	M	Sec	DF	1	29	7	44	7	16	7	15	2	6
P853	8	F	Sec	DF	23	68	49	51	7	15	8	9	3	0
P856	13	F	Sec	DF	3	24	7	18	5	7	5	15	2	6

S1. Cont.

Patient ID	Age	Sex	Type of Infection	Clinical Diagnosis	Human Leukocyte Antigen (HLA)									
					A*	AA*	B*	BB*	C*	CC*	DRB1*	DDRB1*	DQB1*	DDQB1*
P861	8	M	Sec	DHF	2	31	38	44	12	16	7	13	2	6
P862	13	F	Sec	DHF	1	23	50	57	4	6	7	11	3	ND
P863	8	M	Sec	DHF	24	68	15	39	2	7	13	16	3	6
P864	10	M	Sec	DHF	2	24	44	50	5	6	7	15	2	6
P865	3	F	Sec	DHF	2	ND	15	51	2	16	1	1	5	ND
P866	3	F	Sec	DHF	2	24	15	44	5	16	13	0	3	6
P869	0,6	F	Prim	DHF	2	29	50	78	6	16	3	13	2	6
P871	7	F	Sec	DHF	3	68	ND	ND	3	4	3	11	3	4
P872	8	F	Sec	DHF	25	31	18	52	12	15	1	16	3	5
P873	8	F	Sec	DHF	3	24	7	14	7	8	11	15	3	6
P875	6	F	Sec	DHF	1	2	35	44	3	5	4	14	3	0
P877	6	M	Sec	DHF	2	68	27	51	2	15	4	7	2	3
P879	7	F	Sec	DHF	2	23	18	50	6	7	3	4	ND	ND
P880	7	F	Sec	DHF	32	33	15	44	2	5	7	15	2	6
P883	7	M	Sec	DHF	1	68	8	15	2	7	3	4	2	3
P902	8	M	Prim	DHF	3	0	7	41	7	17	11	13	3	6
P912	13	F	Sec	DHF	2	31	7	51	7	15	1	11	3	5
P923	16	M	Sec	DHF	ND	ND	50	58	6	ND	7	15	2	6
P933	-	M	Sec	DHF	30	66	42	51	15	17	4	16	3	5
P934	-	F	Sec	DHF	3	33	14	44	4	8	1	7	2	5
P937	41	F	Sec	DHF	2	74	40	53	3	4	4	13	3	3
P940	12	M	Sec	DHF	29	32	39	44	8	16	7	10	2	5
P956	0,8	M	Sec	DHF	24	74	51	ND	ND	ND	15	15	5	6
P961	0,75	F	Sec	DHF	29	66	44	51	15	16	4	4	3	ND
P970	-	F	Sec	DHF	3	11	44	51	ND	ND	7	7	2	6

S1. Cont.

Patient ID	Age	Sex	Type of Infection	Clinical Diagnosis	Human Leukocyte Antigen (HLA)									
					A*	AA*	B*	BB*	C*	CC*	DRB1*	DDRB1*	DQB1*	DDQB1*
P986	-	M	Sec	DHF	2	3	8	15	7	16	16	16	2	3
P992	12	F	Sec	DHF	1	11	ND	ND	7	16	7	15	2	6
P1036	4	M	Sec	DHF	2	23	45	50	6	16	7	13	2	6
P1046	-	M	Sec	DHF	33	66	18	ND	2	6	4	7	2	3
P1053	-	M	Sec	DHF	23	30	15	50	2	4	7	11	2	3
P1064	-	M	Sec	DHF	2	26	ND	ND	7	ND	4	7	3	0
P1065	-	F	Sec	DHF	2	32	7	15	3	7	16	16	5	ND
P1108	-	M	Sec	DHF	23	ND	7	44	4	7	7	15	2	6
P1110	-	F	Sec	DHF	2	ND	44	53	4	ND	13	0	2	3
P1123	-	M	Prim	DHF	3	26	27	ND	2	2	ND	ND	ND	ND
P1131	-	F	Sec	DHF	24	74	ND	ND	7	16	ND	ND	ND	ND
P1147	-	F	Sec	DHF	2	33	15	56	14	ND	1	8	4	5

-, unkown; F, female; M, male; Prim, primary infection; Sec, secondary infection; DF, dengue fever; DHF, dengue hemorrhagic fever; ND, no defined.
